# Medical sequencing of *de novo* ectodermal dysplasia in identical twins and evaluation of the potential eligibility for recombinant EDA therapy

**DOI:** 10.15171/joddd.2017.025

**Published:** 2017-09-20

**Authors:** Adriana Modesto, Catherine Ventura, Kathleen Deeley, Deborah Studen-Pavlovich, Alexandre R. Vieira

**Affiliations:** ^1^University of Pittsburgh School of Dental Medicine, Department of Pediatric Dentistry, Pittsburgh, PA, USA; ^2^University of Pittsburgh School of Dental Medicine, Department of Oral Biology, Pittsburgh, PA, USA

**Keywords:** Ectodermal Dysplasia, EDA, Mutation

## Abstract

The purpose of this study was to test two 8-year-old identical twins with ectodermal dysplasia (ED) and their unaffected parents for the presence of mutations in the EDA gene with the hypothesis that they might be carrying a de novo mutation in EDA and potentially eligible for recombinant EDA therapy. DNA was extracted using saliva samples obtained from the identical twin girls and both parents. PCR products of Ectodyplasin A (*EDA*), Ectodysplasin Receptor (EDAR), Ectodysplasin Receptor Associated Death Domain (*EDARADD*), and Connexin-30 (*GJB6*) were sequenced by the Sanger method and the results analyzed using a reference sequence. Exons and exon-intron boundaries of *EDA, EDAR, EDARADD*, and *GJB6* were sequenced in both parents and the affected identical twin pair. No mutations were detected in *EDA* or *GJB6*. Genetic variants located in the intron of *EDAR* were found but determined to be non-contributory to the twins’ ED. A microsatellite polymorphism was detected in all four subjects in exon 4 of the *EDARADD* gene but determined not to be causal to the ED. There was a silent mutation detected in exon 6 of the *EDARADD* gene of both the daughters and their unaffected mother but also unlikely to be the cause of ED. These results suggest that ED of the subjects is caused by a *de novo* mutation in a gene not studied here. It is likely these subjects and their future offspring would not benefit from the development of recombinant EDA replacement therapy.

## Introduction

Ectodermal dysplasia syndromes (ED) refer to a group of heterogeneous, inherited disorders characterized by abnormal development of tissues and organs derived from the ectoderm. The incidence is 1 in 100,000 people. There are over a hundred types with variable expression of the effects to the primary and permanent dentitions, skin, sweat glands, hair, nails and other ectodermal structures.^1^ The majority of the subjects affected by the condition have normal brain development and life expectancy. The two major types of ED syndromes are described as hypohidrotic and hidrotic, depending on sweat gland function. Hypohidrotic ED (HED) is the most common form and is named for the absence of sweat glands. Dry skin, eyes, airways and mucous membranes can occur as a result of impaired development of exocrine glands.^2^ In addition to the hypohidrosis seen in HED, hypotrichosis and severe hypodontia are usually present.^3^

**Figure 1:**
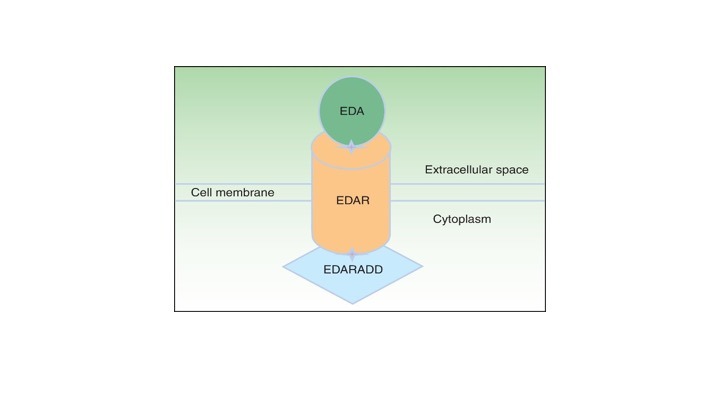
Ectodysplasin-EDAR-EDARADD pathway [modified from (5)].

ED can be inherited by multiple Mendelian pathways: X-linked recessive, autosomal dominant, autosomal recessive, or *de novo*.^4^ Although there are multiple patterns of inheritance, the genetic mutation most commonly occurs in the *EDA* (Ectodysplasin A) gene complex (Figure 1).^5^

Recombinant EDA replacement therapy was successful in dogs and is a promising approach to correct the consequences of inactivating mutations of ectodysplasin A (*EDA*) that lead to X-linked ectodermal dysplasia (ED) in humans.^6^ Identification of potential subjects for a future human trial is an important component of the planning phase of future studies.

The aim of this research hypothesis was to test two 8-year-old identical twin sisters with ED and their unaffected parents for the presence of mutations in the* EDA* gene with the hypothesis that they might be carrying a *de novo* mutation in *EDA* and potentially eligible for recombinant EDA therapy. A complete understanding of the etiology of their ED would be used in conjunction with clinical and environmental features to make appropriate treatment recommendations.

## Case report

**Figure 2:**
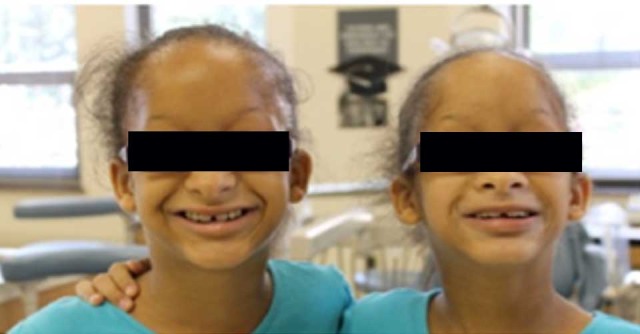
The studied twin sisters.

Identical 8-year-old twin girls were referred by a private dentist in Johnstown, PA, to the University of Pittsburgh School of Dental Medicine (SDM), Department of Pediatric Dentistry (Figure 2). Detailed extraoral and intraoral clinical presentation features were recorded for the twin patients, their parents and one grandparent. The children were confirmed to have ectodermal dysplasia primarily affecting their teeth. There were no other obvious features of ectodermal dysplasia such as sparse hair, dry skin, dysplastic nails, or reports of lack of sweating or fevers when temperature is high. The unaffected mother had agenesis of three third molars (both maxillary and left mandibular) and no other relevant findings. The father was unaffected with an intact dentition. They have a younger brother. The paternal grandmother had agenesis of the left mandibular lateral incisor and the paternal grandfather had no history of tooth agenesis. No information could be obtained for the maternal grandparents.

The subjects and their parents were part of the University of Pittsburgh School of Dental Medicine Dental Registry and DNA Repository project, which is approved by the University of Pittsburgh Institutional Review Board (University of Pittsburgh IRB approval 0609091). Informed consent was obtained from the parents and appropriate assent was obtained from the patients to provide a saliva sample without the use of saliva stimulatory tools. DNA was isolated from the saliva samples collected from the twin children and their parents in Oragene kits (DNA Genotek, ON, Canada) according to the manufacturer's instructions. Primers for the Ectodyplasin (*EDA*), Ectodysplasin Receptor (*EDAR*), Ectodysplasin Receptor Associated Death Domain (*EDARADD*), and Connexin-30 (*GJB6*) genes were obtained from the literature.^7-10^ The primers used are listed in Table 1.

**Table 1:** Primers and PCR conditions used in this study

**Gene**	**Primer Sequence, Forward**	**Primer Sequence, Reverse**	**Annealing Temperature (Celsius)**	**Size of Fragment**
EDA.Exon 4	CCGAGATCGTGCCACTGAACT	CCC CAT CTC CAC CGT TTG AA	53	368
EDA.Exon 1-1	AGAGGTCGTGAACGGCTGAGG	CGCAACTCTAGGTAGCAGCACAAC	54	264
EDA.Exon1–2	GCCTGCTCTTCCTGGGTTTCTT	TCCCTGGTCCTGCCCTCTAAAT	54	400
EDA.Exon2	GCTGGTTTTTTATGTTGGCTATGAC	CCACCATGCCCTACCAAGAAG	52	266
EDA.Exon3	TTTGCAGTGTCTTGGGGATCC	GCAGGGAGAAGAACAAGGAAGAAT	53	346
EDA.Exon5	TCAGGTGAGGGGAAAAGGAAGT	GGGCTGTGAGTGAAAACCGTC	52	240
EDA.Exon6	AGGGGAGAGGGATCAGAATTG	AGGCTGGGTGATTATTTGGAG	50	256
EDA.Exon7	TGCCTCGATTATTCTGACATGTACTG	CCCAAAGCAGGAAGTTAGCCATT	53	300
EDA.Exon8	CCCCACCCTCTCTTTCCTCTCTTC	GGCTGCAACACCAATACACCTCAC	56	412
EDAR.Exon2	TTTGCTGGAAGGCACCTTAT	AGAGGCCAAGAAACAGTCCA	58–62	243
EDAR.Exon3	ACCCCCTTCCTATGTCAACC	CAGGCTCAGGGCAACAAT	56–62	292
EDAR.Exon4	CGGCAAGAGTAGCTTCTGGA	GCAGTATCCATGACCCCTGT	51–63	397
EDAR.Exon5	GTGCTCTCTGCACCAGTCC	GACCGGCTCTTTCCTACACC	52–63	246
EDAR.Exon6	AGCTCTGTGGCAGCGTCT	CCTCTCCTCTTCTGAGCTTTCA	51–62	228
EDAR.Exon7/8	GGAGTCCTGGAGGGAAGACC	AGCATGTGAGAGCAGAAGCA	60	468
EDAR.Exon9	AGAGCAGGGTTGGGCTGAG	GCTAGCCTGTCAGTTCACTCG	51–63	248
EDAR.Exon10	AGGTGCCCAGTAAACACCTG	CGTCTTGCAGGAGAGCTGAT	51–63	400
EDAR.Exon11	CCTGCTGACATGGAGGATTT	CTCAGTTCCCCTCACAGGAG	51–63	234
EDAR.Exon 12	GACCTTCTATTGACTGTGACTTGC	CAGTCTTTTGGCACCACTCA	51-63	461
EDARADD.Exon1-1	GAAAGAACCACAAACCAAACC	TGCCTTCAC ACATAAGAACAG	53-54	452
EDARADD.Exon1-2	AGGTACCGAGGGACGCGC	GGCCTCGATAGCCCTGCG	61-63	323
EDARADD.Exon2	GATTACAGCATGAGCTACCTC	CCAGGGAAGTGGGTAAAGCC	53-58	516
EDARADD.Exon3	CCTTGATTTCATTCCTGTCGA	GTCACGAGCTAATCTATGGGCATG	53-58	352
EDARADD.Exon4	ATCCTTAAGAGCAGAGTTTGG	CTGTTTATGATCTAG AAATCCTG	49-52	348
EDARADD.Exon5	GCGCTCAAGGTGCTCGTATTC	TTACAGGCGCCCACCACAACC	59-63	463
EDARADD.Exon6-2	CGTGTCACCCAACGGTGAAAA	CCTCCACAA AACTGCCAG C	57-58	405
EDARADD.Exon6-1	AAAGAAAGAAACGAGCATTCT	CTGTTCCGGAGCAAGAACTC	50-56	387
Cx30	AGCAGGGCAGGGAGT TGAAG	TCAGGTTGGTATTGCCTTCTGG	57	1008
Cx30.3	CAATCGCACCAGCATTAAGGG	TGATCTTATCTGCTGATCTCGCAG	57	952

Polymerase chain reaction (PCR) conditions were optimized for the primers using stock DNA and a standard PCR protocol. The standard PCR protocol consisted of an initial denaturation step at 95°C for 30 seconds, followed by 30 cycles of denaturation at 94°C for 45 seconds, with a final extension step at 72°C for 5 minutes. PCR product amplification was confirmed by a 3% ethidium bromide agarose gel.

PCR products were cleaned using the PEG precipitation of PCR product protocol. The samples were prepared for sequencing by the Sanger sequencing method used at the Genomics and Proteomics Core facility at the University of Pittsburgh. Microsequencing was completed by capillary electrophoresis using either an Applied Biosystems 3130 or 3730 XL DNA analyzer. The Applied Biosystems sequence software was used for lane tracking and first pass base calling. The sequences were analyzed for mutations by comparing the obtained sequence with a reference gene sequence. Sequencing results were also studied in relation to the clinical presentations of the twin patients and their parents.

Exons and introns from *EDA*, *EDAR*, *EDARADD* and *GJB6* were sequenced for both parents and the affected daughters. No mutations were detected in *EDA* or *GJB6*. Genetic variants located in the intron of *EDAR* were found but determined to be non-contributory to the twins’ ED. A microsatellite polymorphism was detected in all four subjects in exon 4 of the *EDARADD* gene but determined not to be causal to the ED. There was a silent mutation detected in exon 6 of the *EDARADD* gene of both daughters and their mother. This mutation was not found in the father and was unlikely to be the cause of the ED. The base pair T replaced a C but translated to the same amino acid, aspartic acid. This silent mutation has already been reported in the literature and is unlikely to be responsible for ectodermal dysplasia.

## Discussion

Based on clinical presentations, as well as the genetic analysis of the twin girls and their parents, an X-linked recessive pattern of inheritance can be ruled out as a mode of inheritance. There were no mutations found in the *EDA* exons commonly associated with X-linked recessive inheritance. These data confirm that the inheritance pattern is most likely not X-linked recessive.

There were no mutations found in the *EDAR*, nor were *EDARADD* exons reported in conjunction with autosomal dominant and recessive forms of ED. No mutations were detected in *EDA* or *GJB6*. These findings cannot confirm an autosomal dominant or recessive transmission either. According to this initial genetic analysis, the ED mutation is probably located on another gene not examined in this family. An autosomal dominant, or recessive, *de novo* genetic mutation may be located in another pathway involved in ectodermal development in genes such as *MSX1*^11^ or members of the WNT family.^12^ It is likely that the ED of these subjects is caused by a *de novo* mutation in a gene not studied yet. Clinically, the case presented here has the primary features of an ectodermal dysplasia affecting teeth and is comparable to previous reports that identified mutations in *EDA*, *EDAR*, *EDARADD* or *GJB6*.^7‒10^ Aside from the challenges of rehabilitating extensive areas of the arches with congenitally missing teeth, the lifetime prognosis of the condition in the affected children is unlikely to be different from any other similar cases.^5^

Additionally, analysis of these genes and others is necessary to confirm the etiology of ED in the twin's patients. Further research in this area would include sequencing genes outside of the EDA complex less commonly associated with ectodermal dysplasia syndromes. These genes would include *MSX1* or genes in the WNT pathway.

## Conclusion

1. Four different genes commonly associated with ED were eliminated as the location of the disease-causing mutation. These results suggest that the ED of the subjects is caused by a *de novo* mutation in a gene not studied here. Aside from defining the ectodermal dysplasia of these patients as primarily affecting the teeth, we are not able to be more precise.
2. It is likely that these subjects and their future offspring would not benefit from the development of recombinant EDA replacement therapy.
3. When the precise sporadic mutation is confirmed, patients with ED can be provided with a diagnostic target for prenatal management. In confirming the genetic etiology, the patients and their parents may more readily accept the situation in knowing the cause. This acceptance may help them better live with the disorder.

## Acknowledgments

We are indebted with the family that participated.

## Authors’ contributions

AD designed the study, obtained funds, and critically revised the submitted version of the manuscript. CV performed experiments, interpreted data, and wrote first draft of the manuscript. KD performed experiments, interpreted data, and critically revised the submitted version of the manuscript. DSP obtained funds, helped with design, and critically revised the submitted version of the manuscript. ARV designed the study, interpreted the data, and wrote final draft of the manuscript. All authors have read and approved the final version of the manuscript.

## Funding

The Dental Registry and DNA Repository is supported by the School of Dental Medicine. Additional funds were provided by the Department of Pediatric Dentistry, University of Pittsburgh School of Dental Medicine.

## Competing interests

The authors declare no competing interests with regards to authorship and/or publication of this article.

## Ethics approval

The University of Pittsburgh School of Dental Medicine Dental Registry and DNA Repository project is approved by the University of Pittsburgh Institutional Review Board (University of Pittsburgh IRB approval 0609091).

## Consent to publish

Subjects provided written consent for having their pictures published in the scientific literature and used for educational purposes.
